# Acceptability of Traditional Chinese Medicine in Chinese People Based on 10-Year's Real World Study With Mutiple Big Data Mining

**DOI:** 10.3389/fpubh.2021.811730

**Published:** 2022-01-11

**Authors:** Yan Guo, Tengjiao Wang, Wei Chen, Ted J. Kaptchuk, Xilian Li, Xiang Gao, Jiahui Yao, Xudong Tang, Ziming Xu

**Affiliations:** ^1^Xiyuan Hospital Affiliated to China Academy of Chinese Medical Sciences, Beijing, China; ^2^Big Data Center for Arts and Sciences, Peking University, Beijing, China; ^3^Key Laboratory of High Confidence Software Technologies, Ministry of Education and School of Electronics Engineering and Computer Science (EECS), Peking University, Beijing, China; ^4^Global Health and Social Medicine, Harvard Medical School and Beth Israel Deaconess Medical Center, Boston, MA, United States; ^5^Institute of Social Science Survey, Peking University, Beijing, China

**Keywords:** traditional Chinese medicine, TCM, data mining, big data, social review, China

## Abstract

In the past decades, numerous clinical researches have been conducted to illuminate the effects of traditional Chinese medicine for better inheritance and promotion of it, which are mostly clinical trials designed from the doctor's point of view. This large-scale data mining study was conducted from real-world point of view in up to 10 years' big data sets of Traditional Chinese Medicine (TCM) in China, including both medical visits to hospital and cyberspace and contemporaneous social survey data. Finally, some important and interesting findings appear: (1) More Criticisms vs. More Visits. The intensity of criticism increased by 2.33 times over the past 10 years, while the actual number of visits increased by 2.41 times. (2) The people of younger age, highly educated and from economically developed areas have become the primary population for utilizing TCM, which is contrary to common opinions on the characteristics of TCM users. The discovery of this phenomenon indicates that TCM deserves further study on how it treats illness and maintains health.

## Introduction

Along with rapid development of modern medicine, the role of traditional Chinese medicine in health care has received worldwide medical attention (1–5). We found some interesting and important contradictory phenomenon during the development of TCM in China that may be summarized as “More Criticisms vs. More Visits”: (i) the intensity of criticism increased by 2.33 times over the past 10 years, while the actual number of visits increased by 2.41 times in China. (ii) Surprisingly, young, highly educated people, and living in economically developed areas have become the primary characters of population utilizing TCM. This phenomenon subverts the wide accepted thinking that only older people, those lack of modern medical knowledge, or people in economically underdeveloped areas tend to choose TCM treatment. The above conclusions are verified by different data sources using a variety of machine learning methods as follows, indicating prospective clinical application of TCM in China. In order to thoroughly study this phenomenon, we conducted a series of large-scale data analysis and mining experiments on two types of data sets. The details are as follows: Medical visit data for 10 years in China: National statistical data of TCM industry are obtained from National Bureau of Statistics of the People's Republic of China (NBSPR) from 2004 to 2014. In addition to the macroeconomic data of the NBSPR, Xiyuan Hospital of China Academy of Chinese Medical Sciences (also called “Xiyuan Hospital”) contribute a total of 13 million electronic medical records for 10 years for this research. Xiyuan Hospital is a WHO cooperative hospital and one of the most prestigious Chinese medicine hospitals in China. These invaluable medical records cover widespread sources of patients, containing 34 provincial-level administrative units in China and more than 20 countries and regions except China. Cyberspace and social survey data: the public opinion of TCM in cyberspace is obtained from Tianya Forum (the most influential comprehensive network community in China). We extracted a total of 3 billion posts and 20 million users' information involved during over 10 years since 2004. The public opinion of TCM in real world comes from Chinese Family Panel Studies (CFPS), the most influential social survey project in China. Science has reported CFPS in 2010, “60,000 respondents in 25 provinces-making the survey the largest undertaking of its kind in the developing world” (6). In CFPS, we set up a number of questionnaires for the medical treatment of TCM which can comprehensively and objectively reflect the medical viewpoints and crowd characteristics of the Chinese people. In the light of time sequence, we integrate all data into a unified big data platform, which is available for managing and analyzing structured and unstructured data. On this platform, large-scale experiments are conducted on 100-node servers. Following key methods are carried out: (i) topic tracking-active learning method (7) and topical phrases learning method (8) were employed to detect the posts related to TCM topics; (ii) topic mining—we utilize LDA topic mining method (9, 10) to explore the topic categories in posts; (iii) sentiment analysis–opinion aware knowledge graph (11) and sentiment oriented maximum entropy classification method (12) are used to calculate the posters' opinions on TCM; (iv) factor analysis–Lasso (Least Absolute Shrinkage and Selection Operator) Regression (13) and Bayesian network (14) are employed to calculate the dependencies between the growing visits of TCM and the major factors. The above big data platform and machine learning methods can support us to do such objective quantitative analysis: (i) How intense is the criticism of TCM, and the impacts on the actual TCM visiting population? (ii) What are the primary characteristics of people willing to try TCM treatments? (iii) What are the main points of view that people support and oppose TCM?

## Results

### How Intense Is the Criticism of TCM and the Impacts on the Actual Population Making TCM Visits?

**Figure 1:**
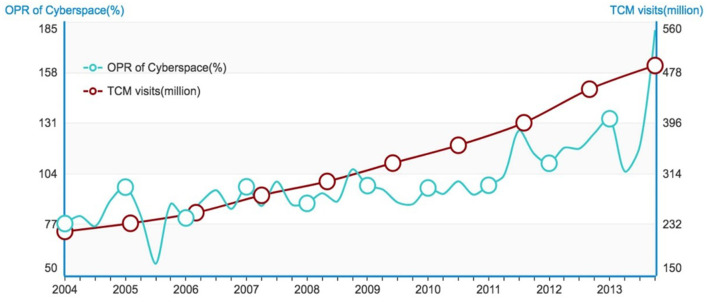
OPR of Cyberspace vs. TCM visits.

We apply topic tracking to extract all posts related to TCM topic from the 3-billion-Tianya-data set. Then a training set is produced using the sentiment oriented maximum entropy classification method (12) to generate an Opinion-aware Knowledge Graph (OKG) (11). And we apply OKG to determine the opinions of the posts about TCM. Posts are classified into three categories according to their opinions: positive, negative and neutral. The intensity of the posts in cyberspace is illustrated in terms of Opinion Polarity Ratio (OPR). In addition, the contemporaneous trends of the medical visits are calculated through the statistical data from NBSPR during 10 years from 2004 to 2014. From the experimental results, a contradictory phenomenon can be clearly identified: Although the intensity of criticisms (quantified by OPR) increased by 2.33 times over the past decade, the actual number of visits has increased annually, with an increase of 2.41 times in 10 years as shown in Figure 1.

### What Are the Characteristics of Populations Utilizing TCM Treatments?

**Figure 2:**
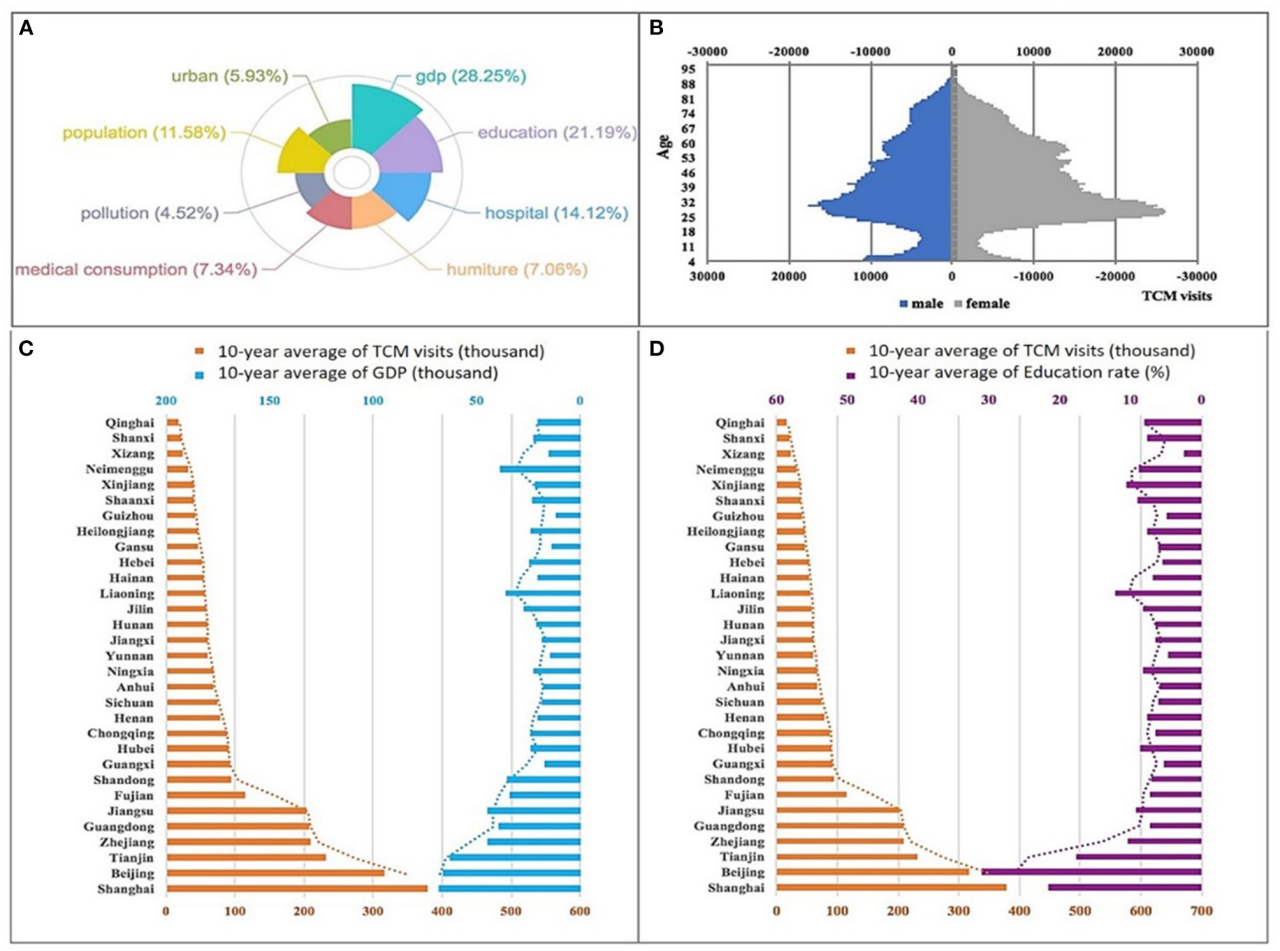
The characteristics of populations utilizing TCM treatments. **(A)** The factor analysis results of Lasso Regression on the NBSPR data. **(B)** The age distribution of Xiyuan Hospital's visit population. **(C)** The relationship analysis between GDP and TCM visits. **(D)** The relationship analysis between education rates and TCM visits.

It is thought that older people, lack of modern medical knowledge and people in economically underdeveloped areas tend to choose TCM treatments. However, we obtain completely opposite results by data mining. In order to objectively and accurately identify the characteristics of people who adhere to TCM treatments, we explore all the key factors that may affect TCM visits in NBSPR and CFPS (The details of all factors can be referenced to Supplementary Table S1). Typical factor analysis methods, Lasso Regression (13) and Bayesian Network (BN) (14), are used to analyze the relationship between these factors and TCM visits. A similar result is obtained from both Lasso Regression and BN: there is a strong correlation between finance, education and the number of TCM visits in a same area (see Supplementary Figure S2). Figure 2A shows the impact coefficients of various factors on the visits of TCM analyzed by Lasso Regression according to the data from NBSPR (see Supplementary Figure S1; Supplementary Table S2 for detailed results). The factors include Gross Domestic Product (GDP), education, hospital infrastructure, medical consumption, pollution levels, temperature and humidity and so on of all provinces and municipalities. The larger the coefficient, the greater the impact of the factor. GDP and education are the two most influential factors. Moreover, by analyzing the factors and their conditional dependencies via a directed acyclic graph represented by Bayesian Network, developed economy and high education have the strongest correlation on the number of TCM visits (see Supplementary Figure S2; Supplementary Table S3). Figures 2C,D illustrate the influence of different levels of GDP and education on the number of visits, respectively. The people with highly educated and from economically developed areas are more likely to be treated by TCM. In addition to the macro-data analysis of the NBSPR, a detailed analysis of the actual visits data contributed by Xiyuan Hospital are realized. Figure 2B depicts the age distributions of Xiyuan Hospital's visit population. It is shown that young people (25–35 years old) with modern science education have become the primary group utilizing TCM treatment! It is surprised to find that the “Little Emperor Group” (15), aged 0–8, has also become a major group utilizing TCM treatment. Thus it can be seen that young parents have high confidence in treating their children with TCM. From the above experimental results, we can draw the conclusion that age, education and economy are the key factors influencing TCM visits. And people of young, highly educated and economically developed areas have become the mainstay of TCM practitioners.

### What Are the Main Reasons That People Support or Oppose TCM?

**Figure 3:**
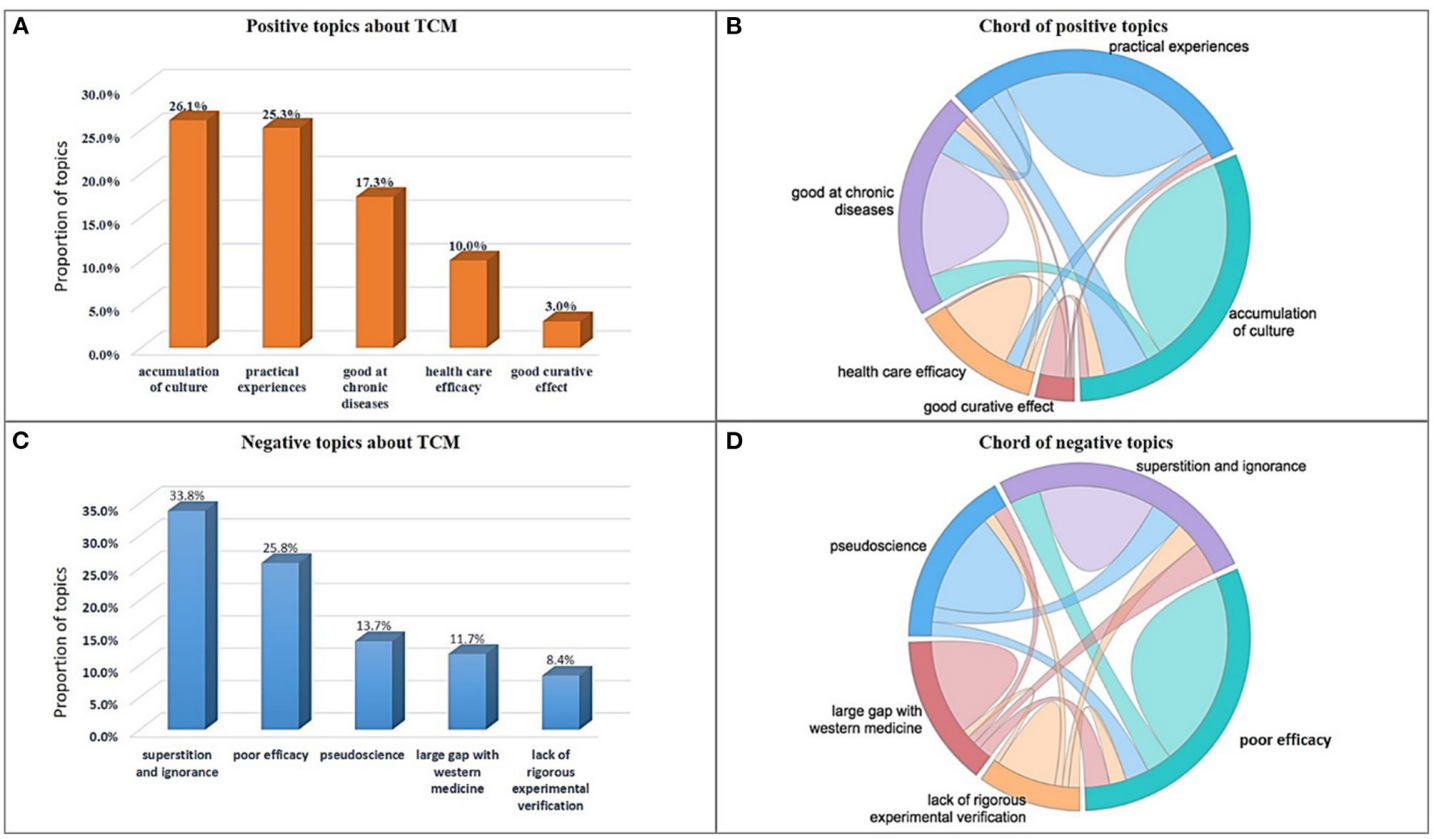
The top five topics about TCM in cyberspace. **(A)** The top five positive topics. **(B)** The co-occurrence relationships between these five positive topics. **(C)** The top five negative topics. **(D)** The co-occurrence relationships between these five negative topics.

In addition to the medical visits, cyberspace has generated a huge amount of TCM related experience sharing data (Tianya) in the past 10 years. People's opinions to TCM are implied in these posts. By using sentiment analysis, posts have already been classified into three categories according to their opinions to TCM: positive, negative and neutral. Topics are extracted from positive posts and negative posts respectively by exploring topic mining methods (9, 10). Each top five of all the topics are displayed in Figure 3.

Figure 3A shows the positive topics of the top five. The main points people endorse for TCM include the accumulation of culture, years of practical experience, effectiveness with chronic diseases, natural health care efficacy and good curative effect. The co-occurrence relationships of these five positive topics are shown in Figure 3B. Figure 3C shows the top five negative topics. The criticisms focus on superstition and ignorance, pseudoscience, poor efficacy, large gap with western medicine, and lack of rigorous experimental verification. The co-occurrence relationships of these five negative topics are shown in Figure 3D (The details of the topics are shown in Supplementary Table S4). People's attitudes toward TCM come from inherent ideas or viewpoints of modern medicine (2, 16). They lack quantitative analysis of the general public opinions. For the first time, the large-scale data analysis has been adopted to calculate people's attitudes toward TCM comprehensively and systematically in this study. The results further verify the contradictory phenomena in this paper.

## Discussion

Traditional Chinese Medicine is the general name of all ethnic medicines in China, with a long history, systematic theory and unique techniques. It is a medical system that reflects the Chinese nation's understanding of life, health and disease. During the past 10 years, the number of online visits to TCM content section has doubled, breaking the stigma that only the elderly, people lacking modern medical knowledge and economically underdeveloped areas will be loyal to Traditional Chinese Medicine in treating diseases and maintaining health. In fact, although more interviews also contain many criticisms, the analysis results still show obvious correlation between economy, education and TCM Treatment, that is, higher the economic and education level, more the acceptance of TCM treatment. The young people aged 25–35 years have become the largest audience group of TCM treatment, exceeding the elderly group in number. In addition, children aged 0–8 is another rising group amazedly, which might be due to their parents aged about 25–35, the number 1 audience of TCM treatment. In addition, Traditional Chinese Medicine has advantages in treating and rehabilitating chronic diseases, based on its theory of wholism and treatment according to syndrome differentiation.

The two sides of things evaluation are always accompanied. Although Traditional Chinese Medicine has made continuous achievements in recent years, especially shown favorable efficacy in fighting for COVID-19 prevalence, negative evaluation will still be an indispensable part, which in return promotes the modernization process of TCM. With the improving economy and expansion of educated popularization, the stereotype of TCM still exists, such as uncertain effectiveness, lack of support of modern scientific experiments, pseudoscience and metaphysics, however, more and more clinical and experimental studies with relatively good and scientific design have come into view, and shown favorable effects on many diseases, especially chronic diseases. And the disease treatment mode, especially those of chronic diseases, has turned to the biological social psychological mode, which is more in line with TCM theory. In the process of spreading TCM, it is absolutely not wise to ignore the negative evaluation. Encouraged by positive evaluation, we should accept criticism and analyze its shortcomings, provide more scientific and objective proofs with modern technology and equipments, to serve the people's healthcare worldwidely.

## Materials and Methods

Although it is widely believed that TCM is full of empirical methods and lack of modern scientific verification, the discovery of the above phenomenon shows that the efficacy of TCM deserves further research. Furthermore, the analysis about the psychology of people's medication selection in modern society will also become an important research topic.

### Definition and Terms

#### Opinion Polarity Ratio

Opinion polarity ratio is the ratio of the total number of negative Tianya posts on TCM topic to the total number of positive Tianya posts. It is abbreviated as OPR. And OPR(s) on the specific topic s is given by the following formula, where n_+_(s) is the total number of positive Tianya posts on the topic s, and n_−_(s) is the total number of negative Tianya posts on the issues.


(1) $$\begin{array}{l} OPR\left(s\right)=\{\begin{array}{l} \frac{n_{-}\left(s\right)}{n_{+}\left(s\right)},n_{+}\left(s\right)\neq 0\\ \frac{n_{-}\left(s\right)+\delta }{n_{+}\left(s\right)+\delta },n_{+}\left(s\right)=0,0<\delta \leq 1 \end{array} \end{array}$$

In order to deal with the situation of the denominator (the number of positive Tianya posts) being 0, smoothing process is needed in the formula. In the experiments, the smoothing factor δ can be taken from 0 to 1 (excluding 0). Sentiment analysis is applied firstly to obtain the opinion polarities of the Tianya posts, and then calculate the OPR on TCM topics.

### Topic Tracking

Topic tracking can overcome the shortcomings of keyword search and minimize the human labeling efforts. We apply a novel multi-label active learning approach with Support Vector Machines (SVM) (8). The detailed process of SVM-based active learning is as follows. For every iteration, we Train a binary SVM classifiers f based on training data Dl. For each instance x in total dataset Du, use the f to assign classification probabilities. Select a set of samples Ds with the largest score, and update the training set Dl = Dl + Ds. At the same time, this set of samples is discarded from Du. If the loop reaches a predetermined number of times, the algorithm terminates and returns to classifier f, otherwise it repeats training SVM. Applying the trained model. In order to process massive posts, the SVM-based active learning classifier is deployed to each data node to do posts classification concurrently. Finally posts related to traditional Chinese medicine are extracted.

### Sentiment Analysis

Textual information is categorized into two types: fact and opinion. Fact is about entities, events, and the corresponding objective description. Opinion is usually used to describe the emotion toward entities or events, which can be divided into positive and negative positions. Sentiment analysis method is used to calculate the public opinion on TCM.

#### Data Preprocessing

To assist the construction of the data sets, beside the word segmentation in topic tracking, the part-of-speech (POS) tagging job is needed additionally.

#### Training Set Construction

In order to reduce the workload and improve the quality of manual annotation, we run MaxEnt-LDA (17) on data set. MaxEnt-LDA can distinguish entities and sentiment words well by utilizing POS and the co-occurrence information between words. In the labeling process, domain experts who do the labeling job can distinguish these two kinds of words, so as to improve the efficiency and accuracy of labeling.

#### Feature Selection

Entities and sentiment words are beneficial features for sentiment analysis. In addition, the post provides some emoticons and punctuation marks to help people express their feelings.

#### Model Training

IIS algorithm (18) is used to train the maximum entropy classifier for post sentiment classification. The main model of classifier is defined as


(2) $$\begin{array}{l} \text{p}\left(c|d,\lambda \right)=\frac{\exp\left[\sum_{i}\lambda_{i}f_{i}\left(c,d\right)\right]}{\sum_{c^{{\;}^{\prime }}}\exp\left[\sum_{i}\lambda_{i}f_{i}\left(c^{{\;}^{\prime }},d\right)\right]} \end{array}$$

where c is the category, d is the post and f is the feature vector. The symbol λ denotes the feature weight vector. A larger λi means that the i-th feature is considered as a stronger indicator for class c. The feature-weight vector λ can be obtained through classifier training. We construct an Opinion-aware Knowledge Graph (11) by integrating the opinions and targeted entities extracted from the training set produced by sentiment oriented maximum entropy classification method (12) into an existing structured knowledge base, and perform stance detection of the expression on TCM conveyed by the posts by information propagation on the graph.

#### Applying the Trained Model

In order to improve the computational efficiency, the trained maximum entropy classifier and OKG are deployed on Hadoop cluster and stance detection is carried out on each data node. Eventually posts are divided into positive, negative and neutral according to its position. An example of sentiment analysis can be seen in Supplementary Table S6.

### Factor Analysis

Factor analysis method (13, 14) is applied to identify the characteristics of people who adhere to TCM treatment. Because these characteristics also demonstrate the impact of various factors on the visits of TCM. We excavated all the key factors that may affect TCM visits in data from National Bureau of Statistics of the People's Republic of China (NBSPR) and Chinese Family Panel Studies (CFPS). The details of total factors can be referenced to Supplementary Table S1. There we use two typical factor analysis methods, Lasso (Least Absolute Shrinkage and Selection Operator) Regression (LR) (13) and Bayesian Network (BN) (14). Factor Analysis includes two stages: data preprocessing and apply the algorithms to select important features.

#### Data Preprocessing

To assist the training data sets for LR and NB, we first filter data from 2004 to 2014 of 34 provinces. 30 factors are collected, including economic, education, medical, population, environment, weather, social security related and other relevant factors.

#### Applying the Algorithms

The most typical factor analysis methods, Lasso Regression (13) and Bayesian Network (BN) (14) were used to analyze the relationship between these factors and TCM visits. The independent variable is the factors after data preprocessing, and the dependent variable is the number of TCM visits.

SciKitLearn is applied to train several feature selection model including Random Forst, Lasso Regression, and PCA models on a single machine with 10-fold cross validation method to determine the optimal parameters. The relationship coefficients between these factors and TCM visits are learned. Moreover, to further prove the result of feature selection by Lasso Regression, we also analyze the factors and their conditional dependencies via a directed acyclic graph represented by Bayesian Network, to revise the factors which have strong influence on the number of TCM visits. Bayesian Network method is embedded in Weka, and some important parameters such as estimator, searchAlgorithm, initAsNaiveBayes, maxNrOfParents, etc. are verified multiple times to select the optimal parameters.

### Topic Mining

We use topic model to mine topics in 3 billion posts based on the online learning. Topic model can learn the word distributions on topics from the word co-occurrence information in corpus. Specifically, we use its online learning variant10 to deal with the huge number of posts, and adapt it to the dynamic vocabulary of posts.

According to the above method, we dig out the topics on the overall 3 billion posts. Including the top five positive topics and five negative topics, the snippet of all the topics is illustrated in Supplementary Table S4 in Chinese.

### Text Preprocessing

In this paper, many mature natural language processing methods are used for text preprocessing in, topic tracking, stance detection, and topic mining including word segmentation and POS tagging. The following describes how to use these text-preprocessing methods in our work (19).

#### Word Segmentation

There is no natural division between Chinese words. Chinese post sequences need to be segmented into separate words. Taking user IDs, URLs and email addresses appeared in posts into consideration, we first extract these proper nouns by the predefined regular expressions from the posts and then use ICTCLAS tool to segment words (20). Because the segmentation tool has been trained on a smaller dataset beforehand, and the segmentation on posts can be parallelized, we deploy the segmentation tool onto data nodes and perform word segmentation on the big data platform.

#### POS Tagging

We mentioned that there are beneficial features to determine the sentiment of a sentence in sentiment analysis section, including sentiment words (adjectives, verbs and others), emoticons and punctuation marks. So it's necessary to determine each word's grammatical rule in the sentence, such as noun, pronoun, verb, adverb, adjective, preposition, conjunction, interjection, and numeral, date, article and determiner. POS tagging is designed for this purpose (17). We use Stanford POS Tagger (21), which is also available for Chinese, to tag posts. And POS tagging is parallelizable for posts. We carry it out on the big data platform in the distributed way. The POS tagging result can be used for selecting sentiment words. It also provides POS information for the MaxEnt-LDA algorithm, which assists experts with building sentiment analysis training sets.

### Computing Environment

Discovering the difference of public opinion between the real world and cyberspace requires the support of high performance computing cluster. So we build a distributed computing platform by 100-node servers to support the large-scale data experiments. Machine configurations are shown in Supplementary Table S5; Supplementary Figure S3. We integrate social survey data and posts into a unified big data platform, which is available for managing and analyzing structured and unstructured data. Supplementary Figure S5 shows the network configuration information of our cluster. Machines in blue are center servers, machines in orange are name nodes and job trackers, machines in purple run zookeeper to monitor the whole cluster, machines in green are installed with cloud database to restore the computing result, and others are data nodes and task trackers. We firstly train the model on a single machine, and then we deploy the trained model on data nodes in the map-reduce way.

## Data Availability Statement

The raw data supporting the conclusions of this article will be made available by the authors, without undue reservation.

## Author Contributions

TW and YG contributed to the overall study design and paper writing. WC contributed to method design and data analysis. TK and XT contributed to medical consultation and method evaluation. XL contributed to data collection and experiment. JY contributed to social issue analysis. All authors contributed to the article and approved the submitted version.

## Funding

This research is supported by the National Natural Science Foundation of China (Nos. 62176005 and 81774138). Project 2020BD002 supported by PKU-Baidu Fund and Xiyuan Hospital of China Academy of Chinese Medical Sciences.

## Conflict of Interest

The authors declare that the research was conducted in the absence of any commercial or financial relationships that could be construed as a potential conflict of interest.

## Publisher's Note

All claims expressed in this article are solely those of the authors and do not necessarily represent those of their affiliated organizations, or those of the publisher, the editors and the reviewers. Any product that may be evaluated in this article, or claim that may be made by its manufacturer, is not guaranteed or endorsed by the publisher.
