# Supplementary material for: Acceptability of Traditional Chinese Medicine in Chinese People Based on 10-Year's Real World Study With Mutiple Big Data Mining
Source: Front Public Health. 2022 Jan 11;9:811730. doi: 10.3389/fpubh.2021.811730 (PMC8802718; doi:10.3389/fpubh.2021.811730)
Supplement: Supplementary file 1 [file Data_Sheet_1.docx]

**Supplementary Material for**

**Acceptability Of Traditional Chinese Medicine In Chinese People Based On Ten-Year's Real World Study With Mutiple Bigdata Mining**

**This File includes:**

Figures S1-S3

Tables S1-S6

# SUPPLEMENTARY FIGURES


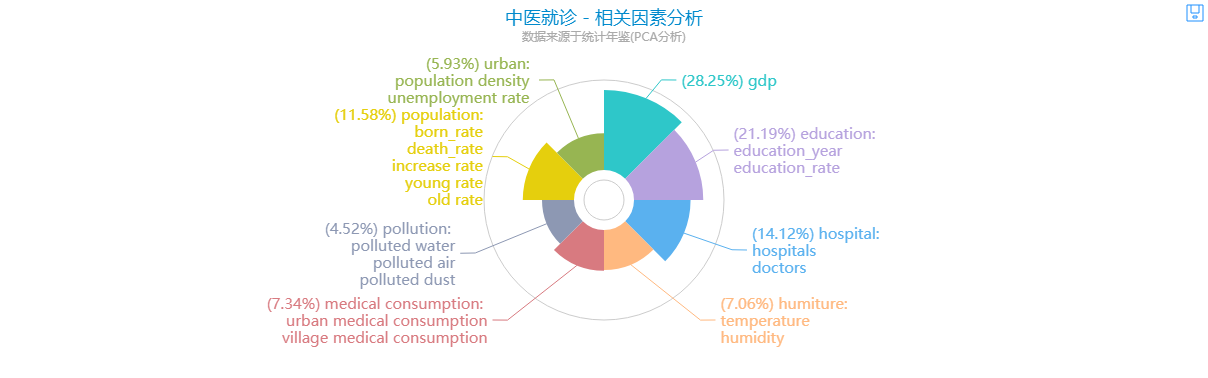


**Fig.S1. Detailed factor analysis result of Lasso Regression on the data of NBSPR**

**Fig. S2 (a) Bayesian network on the data of NBSPR.**

z

**Fig. S2 (b) Conditional probability table of the top three factors.**

**Fig. S3. Network configurations.**

# SUPPLEMENTARY TABLES

**Table S1. All the key factors which may influence TCM visits**

| Integrated factors | Detailed factors | Descriptions |
| --- | --- | --- |
| GDP | GDP(Gross Domestic Product) | A monetary measure of the market value of all final goods and services produced in a period (quarterly or yearly) of time |
| Education | edu_year | Years of education |
|  | edu_rate | Education level, such as undergraduate or graduate |
| Hospital | hospitals | Number of hospitals in a region |
|  | doctors | Number of doctors in a region |
| Humiture (temp-hum) | temperature | Temperature in a region |
|  | humidity | Humidity in a region |
| Medical consumption | urban medical consumption | Urban medical consumption of one province |
|  | village medical consumption | Village medical consumption of one province |
| pollution | polluted water | The extent of water pollution in a region |
|  | polluted air | The extent of air pollution in a region |
|  | polluted dust | The extent of dust pollution in a region |
| population | born rate | Birth rate in a region |
|  | death rate | Death rate in a region |
|  | increase rate | Death rate in a region |
|  | young rate | Population growth rate in a region |
|  | old rate | Population ageing rate in a region |
| urban | population density | Urban population density of one province |
|  | unemployed rate | Urban unemployed rate of one province |
| other | mean life | Mean life in a region |
|  | ocean | Is it coastal? |
|  | altitude | What is the altitude? |

**Table S2. Normalized weights of factors learned by Lasso Regression (NBSPR data)**

| Model  Factor | Lasso Regression |
| --- | --- |
| gdp | 28.25% |
| edu_year | 9.32% |
| edu_rate | 11.87% |
| hospitals | 0.57% |
| doctors | 6.78% |
| temperature | 3.67% |
| humidity | 10.45% |
| outcome_hospital | 0.85% |
| village_outcome_hospital | 6.22% |
| polluted-water | 0.28% |
| polluted-air | 3.67% |
| polluted-dust | 0.57% |
| young_rate | 4.80% |
| old_rate | 5.09% |
| born_rate | 1.41% |
| death_rate | 0.00% |
| natural_increase_rate | 0.28% |
| urban_pop_density | 1.41% |
| urban_unemployed_rate | 4.52% |
| meanLife | 0.28% |
| ocean | 1.13% |
| altitude | 0.57% |

**Table S3. Conditional probability table of the Bayesian Network**

| GDP | 0.33 | 0.32 | 0.26 | 0.08 | 0.01 |
| --- | --- | --- | --- | --- | --- |
|  | 0.09 | 0.13 | 0.21 | 0.35 | 0.21 |
|  | 0.07 | 0.01 | 0.04 | 0.33 | 0.54 |
|  | 0.03 | 0.03 | 0.03 | 0.23 | 0.69 |
|  | 0.04 | 0.04 | 0.04 | 0.04 | 0.83 |
|  |  |  |  |  |  |
| Education | 0.26 | 0.25 | 0.26 | 0.15 | 0.09 |
|  | 0.12 | 0.13 | 0.12 | 0.32 | 0.30 |
|  | 0.02 | 0.02 | 0.02 | 0.21 | 0.72 |
|  | 0.07 | 0.07 | 0.07 | 0.33 | 0.47 |
|  | 0.09 | 0.09 | 0.09 | 0.09 | 0.64 |
|  |  |  |  |  |  |
| Doctors | 0.39 | 0.22 | 0.13 | 0.17 | 0.08 |
|  | 0.21 | 0.21 | 0.16 | 0.21 | 0.21 |
|  | 0.09 | 0.25 | 0.29 | 0.23 | 0.13 |
|  | 0.02 | 0.08 | 0.41 | 0.25 | 0.34 |
|  | 0.03 | 0.03 | 0.13 | 0.31 | 0.70 |
|  |  |  |  |  |  |
| Hospitals | 0.30 | 0.22 | 0.18 | 0.16 | 0.14 |
|  | 0.06 | 0.21 | 0.19 | 0.28 | 0.26 |
|  | 0.10 | 0.13 | 0.22 | 0.22 | 0.33 |
|  | 0.06 | 0.06 | 0.41 | 0.18 | 0.29 |
|  | 0.02 | 0.12 | 0.30 | 0.26 | 0.30 |

**Table S4. Partial topics in Tianya forum posts in Chinese.**


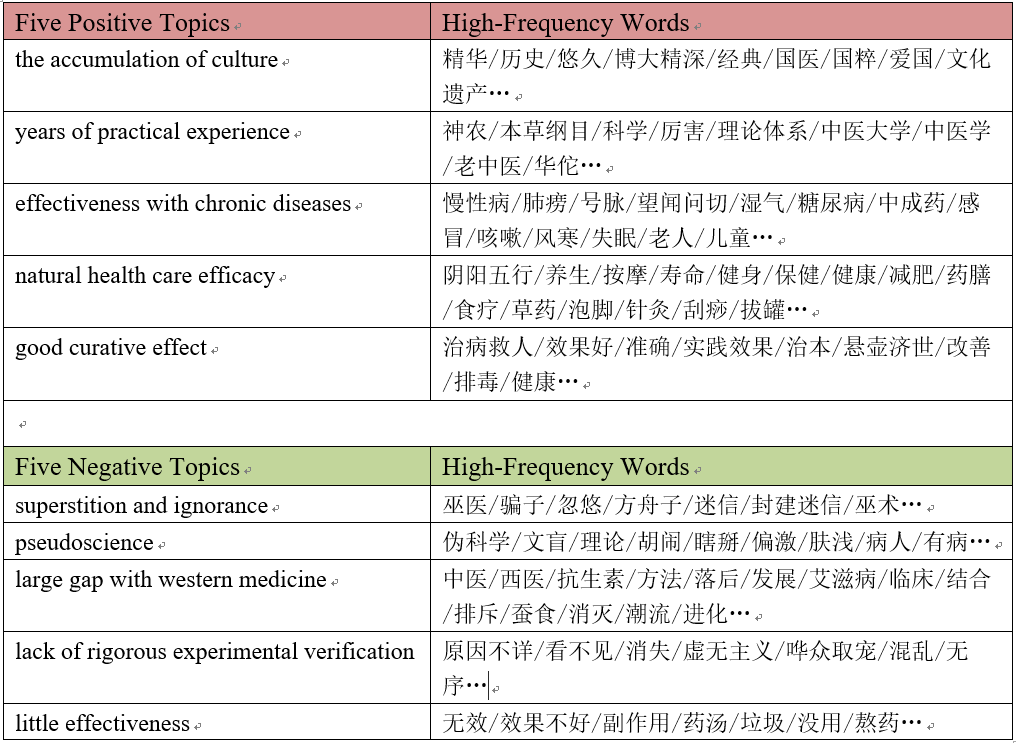


**Table S5. Machine configurations**

| Number of machine | 100 rack-mounted server machine of Dell |
| --- | --- |
| Capacity of single machine | Each machine is equipped with TB level storage space, an 8-core processor and 16 GB memory capacity. |
| Network configuration | The bandwidth within racks is 9 GB per second, and the bandwidth between racks is 6 GB per second. |

**Table S6. The opinion polarity of the sample post**


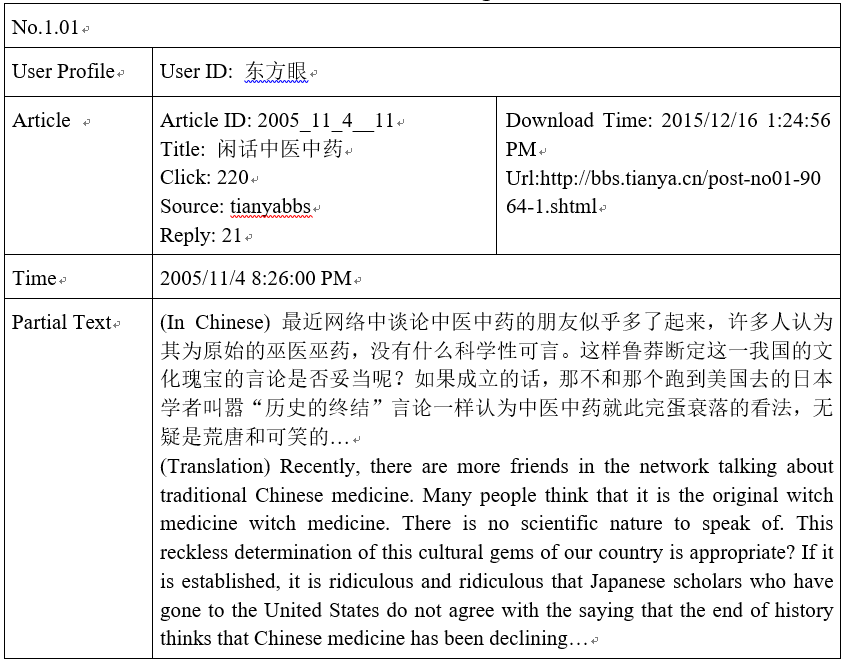


Opinion Analysis Result: 1(positive)
